# The pro-tumorigenic roles of granzyme B: mechanisms and therapeutic implications

**DOI:** 10.3389/fimmu.2025.1733793

**Published:** 2026-01-06

**Authors:** Yubi Zhang, Han Huang, Linjun Xie, Chunhong Li, Xiangyu Zhou

**Affiliations:** 1 Department of Thyroid Surgery, the Affiliated Hospital of Southwest Medical University, Luzhou Sichuan, China; 2 Basic Medicine Research Innovation Center for Cardiometabolic Disease, Ministry of Education, Southwest Medical University, Luzhou Sichuan, China; 3 Department of Pharmaceutical Sciences, School of Pharmacy, Southwest Medical University, Luzhou Sichuan, China

**Keywords:** epithelial-mesenchymal transition(EMT), extracellular matrix (ECM) remodeling, granzyme B(GZMB), pro-tumor, tumor microenvironment(TME)

## Abstract

Granzyme B (GZMB) is an effector molecule primarily expressed by cytotoxic T lymphocytes (CTLs) and natural killer (NK) cells. Historically, GZMB expression levels have served as a marker of immune activity, indicative of the potency of anti-tumor immunity. However, recent evidence increasingly demonstrates that GZMB also exerts immunosuppressive effects within the tumor microenvironment. Beyond CTLs and NK cells, GZMB derived from multiple immune and tumor cells promotes tumor initiation and progression by regulating biological processes such as extracellular matrix remodeling, epithelial-mesenchymal transition, and angiogenesis. This paper summarizes the pro-tumor sources and mechanisms of GZMB, providing a comprehensive understanding of its clinical significance to guide more holistic GZMB-based anti-tumor therapies.

## 1 Introduction

Granzymes are a class of serine proteases with five primary subtypes present in humans, including granzyme A, granzyme B (GZMB), granzyme H, granzyme K, and granzyme M, among which GZMB is the primary effector molecule (1, 2). GZMB is primarily synthesized by cytotoxic T lymphocytes (CTLs) and natural killer (NK) cells and stored within cellular granules. During immune responses, GZMB is delivered into the cytoplasm of target cells via perforin-mediated entry, activating the caspase cascade and inducing programmed cell death (2, 3). Recent studies reveal that beyond classical perforin-dependent cytotoxicity, GZMB possesses perforin-independent functions, including roles in regulating the tumor microenvironment (TME), degrading the extracellular matrix (ECM), modulating inflammatory responses, and influencing other biological processes (4–6).

GZMB is initially synthesized as a zymogen that includes a signal peptide, a pro-domain, and an active enzyme domain. The human GZMB precursor is composed of 247 amino acids. Proteolytic cleavage of the pro-domain results in the formation of the active mature enzyme, which consists of approximately 242 amino acids. Its three-dimensional structure features a classic trypsin-like fold, composed of two β-barrel domains with an active site situated between them. This active site is characterized by a typical catalytic triad (His57-Asp102-Ser195) essential for the hydrolysis of peptide bonds. Uniquely, the substrate-binding pocket of GZMB demonstrates strict specificity for aspartic acid (Asp), enabling it to selectively cleave peptide bonds that follow Asp residues in target proteins. This specificity facilitates the efficient activation of the apoptotic pathway while preventing the non-specific degradation of other proteins (7–9).

Previous studies have documented GZMB’s anti-tumor effects through classical mechanisms, highlighting its significance in tumor immunity. However, emerging evidence also suggests that its elevated expression may be associated with pro-tumor effects. Consequently, this paper aims to summarize the role of GZMB’s non-classical pathways in tumor initiation and progression, thereby enhancing our understanding of its intricate mechanisms.

## 2 The classical anti-tumor action of GZMB

**Figure 1:**
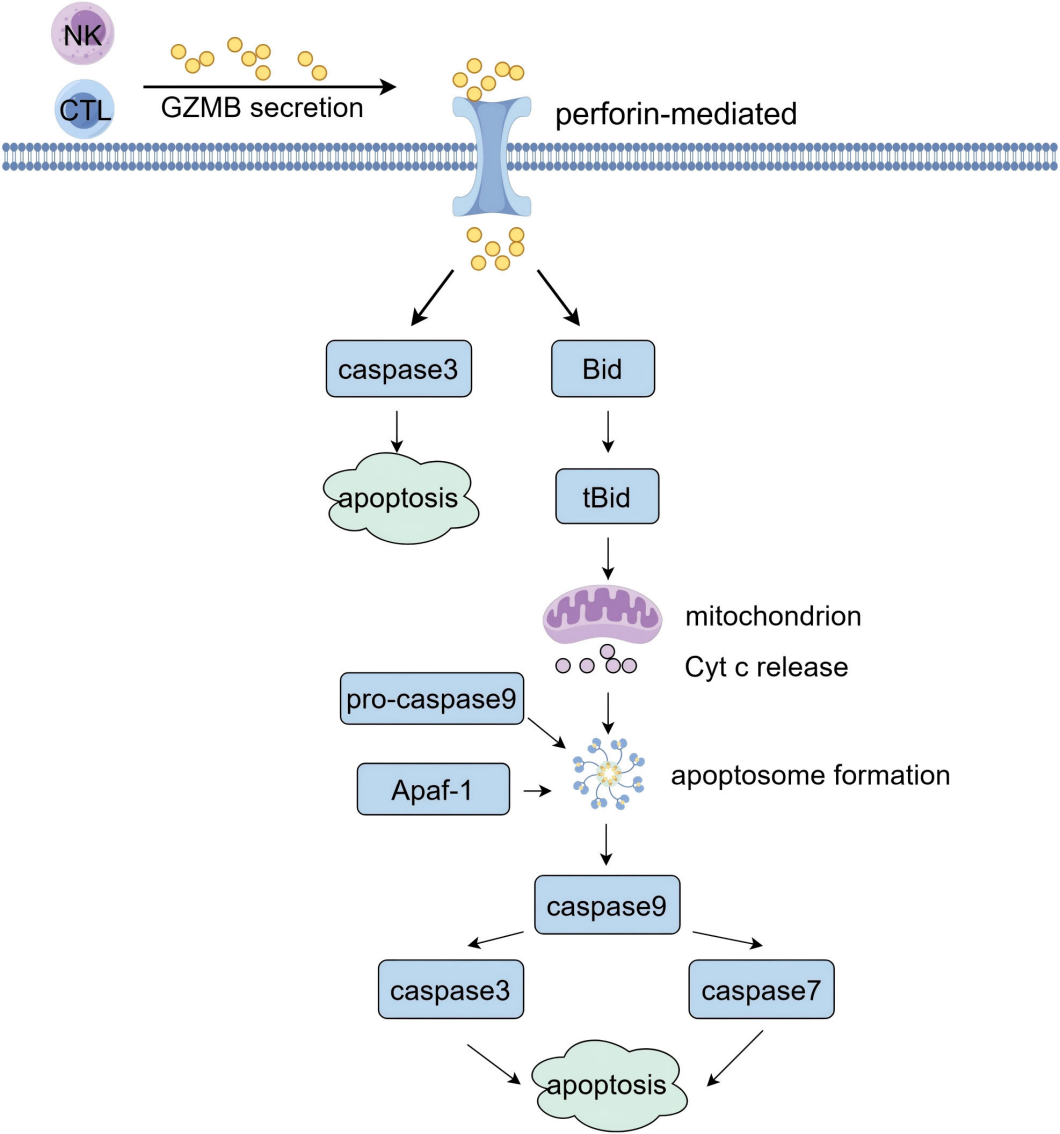
The classical pro-apoptotic mechanism of GZMB.

The primary function of GZMB is mediated through its expression in CTLs and NK cells. Upon recognition of a target cell by these immune cells, GZMB is delivered into the target cell via pores created by perforin in the cell membrane, where it exerts its anti-tumor effects through the cleavage of specific substrates. As depicted in **Figure 1**, GZMB directly cleaves caspase-3, thereby activating the apoptotic pathway. Simultaneously, it cleaves the pro-apoptotic protein Bid into its active form, tBid, which then translocates to the mitochondrial membrane. This translocation results in a loss of membrane potential and an increase in membrane permeability, leading to the release of cytochrome c into the cytoplasm. Cytochrome c subsequently forms a complex with Apaf-1 and the precursor of caspase-9, which initiates the activation of caspase-9. This activation further facilitates the activation of caspase-3 and caspase-7, thereby amplifying the apoptotic signal (3, 10–12).

Extensive research indicates that GZMB expression levels in tumors correlate closely with patient prognosis, with higher expression associated with improved outcomes. In solid tumors, such as lung and breast cancer, GZMB is extensively studied as a marker of anti-tumor immune activity. High expression of GZMB is associated not only with significantly prolonged disease-free survival (DFS) and overall survival (OS) but may also exhibit a negative correlation with the expression of immune-suppressive molecules such as PD-L1 and IDO1 (13–16). In immunotherapy, GZMB expression serves as a biomarker for predicting the efficacy of immune checkpoint inhibitors (ICIs). GZMB PET imaging technology can be employed for early assessment of responses to cancer immunotherapy (17, 18). Recently, nanotechnology has offered novel avenues for the precise delivery and functional regulation of GZMB, aiming to achieve specific targeting and efficient killing of tumor cells through this approach (19, 20). In addition, the positive effects of GZMB are extensively studied in its targeted drug delivery systems. These investigations primarily rely on GZMB expression in CTLs and NK cells. However, immune cells within the TME, such as mast cells and regulatory T cells (Tregs), can similarly influence its expression and function, thereby affecting tumor immune evasion and progression. Nevertheless, most current research has focused solely on its anti-tumor effects, overlooking its potential pro-tumor regulatory roles. This paper will primarily summarize and categorize the “negative effects” of GZMB.

## 3 The pro-tumorigenic sources of GZMB: beyond classical immune cells

Recent findings have expanded our understanding of GZMB production beyond conventional CTLs and NK cells to include Tregs, myeloid-derived suppressor cells (MDSCs), plasmacytoid dendritic cells (pDCs), and even tumor cells themselves, as summarized in **Table 1**. This non-classical production of GZMB contributes to various biological processes including inflammatory responses, regulation of angiogenesis, and ECM degradation, thereby exerting immunosuppressive effects and underscoring its multifaceted role in tumor initiation and progression (5, 6, 21).

**Table 1:** Cellular origins of GZMB-mediated pro-tumor effects.

Cell type	Mechanism	Key stimulatory and regulatory factors	Function	Refs
Regulatory T Cells (Tregs)	Kill NK cells, CD8+ T cells	High Concentration IL-2	Promotes tumor progression	(27–30)
Myeloid-Derived Suppressor Cells (MDSCs)	Kill CD8+ T cells	GM-CSF	Promotes tumor progression	(35, 36)
Plasmacytoid Dendritic Cells (pDCs)	Suppress T cell proliferation	IL-3, IL-10, IL-21	Promotes tumor progression	(39–42)
Regulatory B Cells (Bregs)	Suppress T cell proliferation	IL-21	Promotes tumor progression	(48)
Mast Cells	Degrade ECM proteins	FGF-1,GM-CSF	Promotes angiogenesis	(53)
Tumor Cells	Degrade ECM proteins, Regulate EMT	TGF-β	Promotes tumor invasion	(57–59)

Tregs, a specialized subset of CD4+ T cells, are essential for maintaining immune tolerance within the body. Research has shown that certain Tregs are also capable of secreting GZMB (22–24). They suppress the immune responses of other immune cells *in vivo*, ultimately facilitating immune escape of tumor cells and inhibiting tumor-associated immune responses. This suppression encompasses the killing of autoreactive immune cells, inhibition of B cell proliferation, and suppression of effector T cell functions (25, 26). Numerous studies have illustrated that GZMB is pivotal in this context, serving as a critical element of Treg-mediated immune suppression. For instance, it has been demonstrated in murine models that GZMB produced by Tregs promotes melanoma progression and metastasis. Notably, in the absence of CD8+ T cells, Treg-derived GZMB still enhanced lung metastasis in these models, indicating its ability to promote tumor metastasis independently of cytotoxic T cells (27). Further research has shown that GZMB+ Tregs can directly induce apoptosis of effector T cells through both perforin-dependent and -independent pathways (22, 28), implying that GZMB produced by Tregs undermines the capacity of effector cells to eradicate tumors by killing NK cells and CD8+ T cells (29). A pertinent question arises regarding the role of GZMB when both Tregs and reactive T cells coexist. Czystowska et al. discovered that interleukin-2 (IL-2) plays a crucial mediating role in this dynamic. At lower concentrations (150 IU/mL), Tregs neither express GZMB nor perforin, whereas at elevated concentrations (1000 IU/mL), Tregs express both molecules, which enables them to induce death in reactive T cells while simultaneously remaining resistant to cell death themselves, even when perforin activation is inhibited. This suggests an operation independent of perforin (30). Furthermore, Azzi et al. found that activated Tregs express the serine protease inhibitor 6 (Spi6) intracellularly, which plays a critical role in Treg homeostasis by protecting activated Tregs from GZMB-mediated damage (31).

Beyond Tregs, investigations have demonstrated the expression of GZMB in MDSCs, a group of bone marrow-derived inhibitory cells that include precursors to dendritic cells, macrophages, and granulocytes. Under pathological conditions, the maturation of these cells is hindered, ultimately resulting in the emergence of immunosuppressive MDSCs associated with poor tumor prognosis. MDSCs are believed to play significant roles in tumor survival and metastasis through various mechanisms, including the induction of Tregs, production of interleukin-10 (IL-10), and modulation of NK cells (32–34). Dufait I et al. revealed that immature dendritic cells could express GZMB following stimulation with granulocyte-macrophage colony-stimulating factor (GM-CSF), subsequently killing CD8+ T cells via a cell-contact and perforin-dependent mechanism (35). Further research has shown that GZMB derived from mouse MDSCs promotes the growth of melanoma tumor cells and alters the proportion of CD8+ T cells, with the specific mechanisms requiring further exploration (36).

Human pDCs, a specialized subset of dendritic cells that bridge innate and adaptive immunity, also significantly contribute to tumor-promoting GZMB activities (37, 38). Research has shown that pDCs secrete substantial amounts of GZMB when stimulated by IL-3 and IL-10, with IL-10 mediating this process via the JAK1-STAT3/5 pathway. GZMB+ pDCs suppress T-cell proliferation through a perforin-independent mechanism, thereby significantly contributing to tumor immune evasion (39). Furthermore, Bratke K et al. observed that GZMB expression was upregulated by stimulation with IL-3, specifically enhancing its expression without affecting other granzymes or perforin. They also demonstrated that TLR7/9 ligands significantly suppressed its expression in pDCs (40). Beyond the involvement of IL-3 and IL-10, Karrich JJ et al. identified that IL-21 also promotes GZMB secretion. Human pDCs stimulated with IL-21 exhibited markedly elevated expression of GZMB, subsequently impairing the proliferation capacity of CD4^+^ T cells (41). Fabricius D et al. elucidated this mechanism, demonstrating that pDCs transferred its high levels of GZMB to T cells, where it degraded the zeta (ζ) chain of the TCR in a GZMB-dependent manner. This provides a plausible explanation for how GZMB+ pDCs suppress T cell proliferation. Moreover, inhibitors of GZMB can reverse this response, leading to enhanced T cell proliferation (42).

Previous research has consistently shown that B cell-derived GZMB plays a pivotal role in anti-tumor activity, with patients exhibiting a higher proportion of GZMB^+^ B cells demonstrating improved prognoses (43–45). However, these cells have also been found to potentially exert immunosuppressive effects. Lindner S and colleagues identified that regulatory B cells (Bregs), a specialized subset of B lymphocytes, can secrete GZMB following induction by IL-21. Analogous to Tregs, Bregs primarily perform immunosuppressive functions, often through the secretion of inhibitory cytokines such as transforming growth factor-β (TGF-β) and IL-10 (46, 47). GZMB secreted by Bregs impairs T cell responses by disrupting the ζ chain in their TCR (48). Recently, Zhu JQ and colleagues analyzed single-cell sequencing data from intrahepatic cholangiocarcinoma (iCCA) in the GEO database and found that tumor tissues exhibited a higher percentage of GZMB^+^ B cells, which are chemotactically recruited by tumor cells via the MIF-(CD74+CXCR4) signaling pathway. However, the pro-apoptotic effect of GZMB is suppressed, potentially contributing to tumor progression. The specific mechanisms underlying this phenomenon warrant further investigation (49).

Furthermore, numerous studies have shown that mast cells can also produce GZMB (5, 50–52). Mast cells, which are widely distributed throughout the immune system, play a critical role in allergic reactions and contribute to immune regulation. The implications of mast cell-derived GZMB in immune modulation remain a subject of research. Studies reveal that GZMB^+^ mast cells promote ECM remodeling, an effect that also occurs in tumors. Wroblewski M and colleagues suggested that mast cell-derived GZMB degrades components such as laminin and fibronectin within the ECM, releasing proangiogenic factors (e.g., FGF-1, GM-CSF) from the ECM, ultimately inducing angiogenesis and contributing to a poor prognosis (53). Additional studies have indicated that mast cells themselves can express serine protease inhibitors, providing protection against the effects of both endogenous and locally released GZMB (54, 55).

In parallel, research has demonstrated that certain tumor cells also express GZMB. As early as 2003, during immunohistochemical analysis on paraffin-embedded breast cancer sections, Hu SX and colleagues noted that breast cancer cells could express GZMB alongside endogenous retinoblastoma protein (pRB) expression, though the relationship between the two and their specific functions was not elucidated at that time (56). Subsequent research by D’Eliseo D and others revealed that both bladder and pancreatic cancer cells express GZMB, which enhances tumor invasiveness. Additionally, downregulating GZMB significantly reduced the invasive capacity of these tumors (57, 58). Similarly, it has been demonstrated that colorectal cancer (CRC) cells express GZMB, promoting tumor invasiveness via the epithelial-mesenchymal transition (EMT) pathway (59). These studies collectively indicate that GZMB expression by tumor cells regulates tumor initiation and progression.

Consequently, substantial evidence indicates that a variety of immune cells, including Tregs, MDSCs, pDCs, Bregs, and mast cells, as well as tumor cells themselves, can express GZMB. These non-classical sources of GZMB not only participate in ECM remodeling and EMT but also exert significant pro-tumorigenic effects by killing or suppressing effector T cells and disrupting their receptor signaling. Additionally, cytokines within the TME and endogenous inhibitors within these cells precisely regulate the expression and activity of GZMB, thereby adding further complexity to its role in tumor progression.

## 4 The mechanisms of the pro-tumorigenic action of GZMB

**Figure 2:**
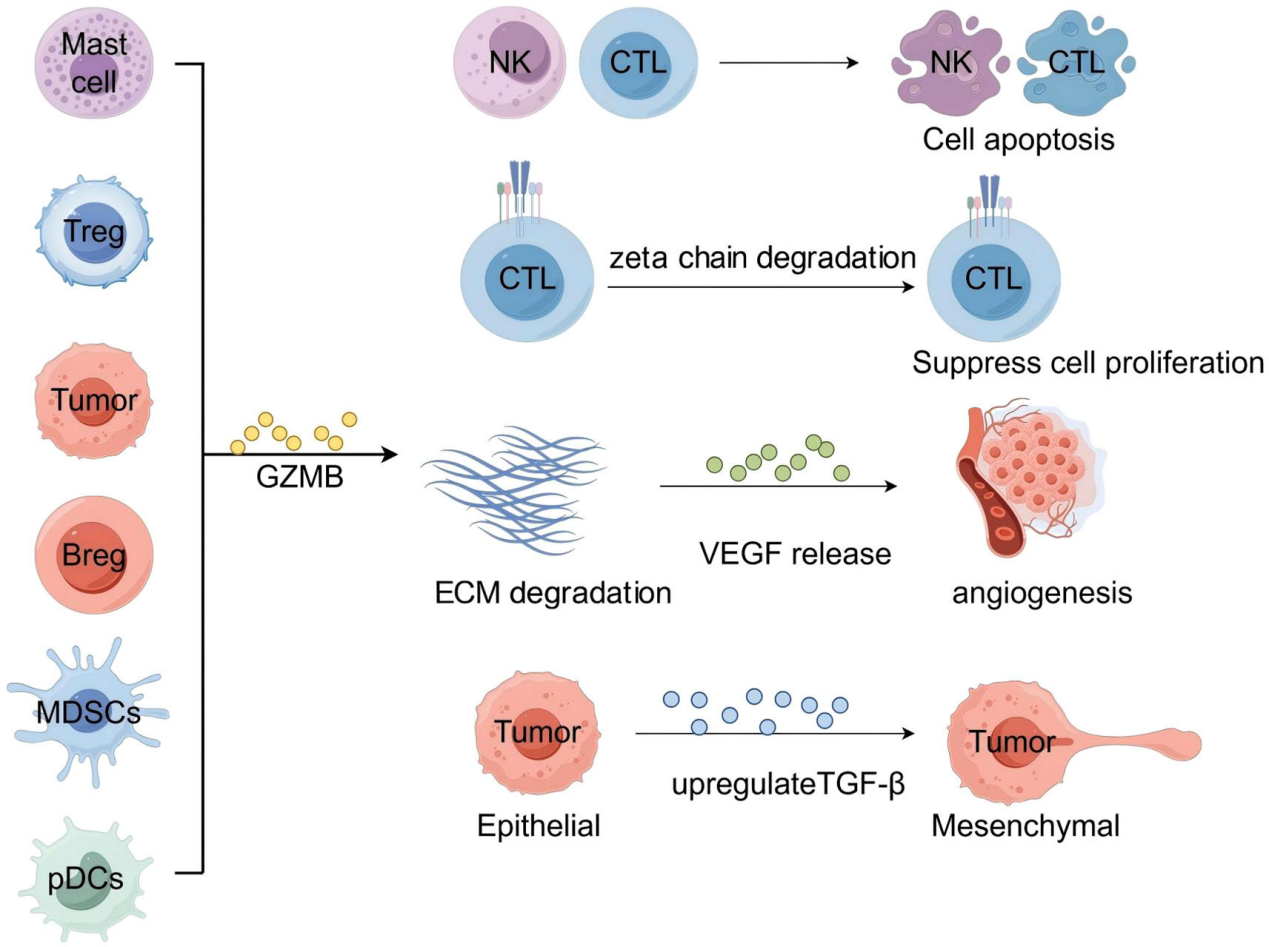
The mechanism of the pro-tumorigenic effect of GZMB.

How does GZMB specifically promote tumorigenesis and progression? Its influence is primarily attributed to its regulatory role within the TME, including the modulation of ECM remodeling, the EMT, tumor angiogenesis, and T cell proliferation and apoptosis, as illustrated in **Figure 2**. This chapter will focus on elaborating these mechanisms.

### 4.1 Extracellular matrix remodeling

As a crucial component of the TME, ECM comprises collagen, elastin, fibronectin, and additional components, engaging in intricate interactions with tumors. The ECM serves not only as a structural scaffold but also regulates cellular functions such as metabolism, polarity, migration, and proliferation through its biochemical and biophysical properties, thus facilitating tumor initiation and progression. The ECM exists primarily in two forms: the interstitial matrix and the basement membrane. The interstitial matrix predominantly connects cells to the matrix, whereas the basement membrane primarily preserves tissue integrity (60–62). As early as 2005, Buzza MS et al. proposed that human GZMB directly cleaves components like fibronectin and laminin, contributing to ECM remodeling (63). By specifically cleaving these proteins, GZMB compromises ECM integrity, thereby facilitating tumor cell invasion and migration. One study indicated that GZMB enhances lymphocyte migration by cleaving components of the basement membrane of the ECM (64). In 2010, research further substantiated the role of GZMB in enhancing tumor invasiveness through ECM remodeling. Studies identified GZMB expression in urothelial carcinoma (UC) tissues, revealing statistically significant correlations between its expression levels and tumor grading, and demonstrating that GZMB remodeled the ECM in UC by cleaving desmoglein, thus enhancing tumor invasive capabilities (57, 65).

Additionally, GZMB may indirectly regulate the ECM by affecting the activity of matrix metalloproteinases (MMPs). MMPs, a zinc ion (Zn^2+^)-dependent family of endopeptidases, primarily function in degrading the ECM. Previous studies have indicated that MMP-mediated ECM remodeling is a critical step in tumor invasion and metastasis (66, 67). Ben-Eltriki M et al. showed that GZMB promoted the release of MMP-1 from gingival fibroblasts in a PAR1- and Erk1/2-dependent manner (68). Further research demonstrated that GZMB is involved in both direct and indirect degradation of the skin ECM following ultraviolet irradiation, where GZMB directly cleaved ECM proteins such as fibronectin and decorin. This degradation subsequently enhances fibroblast expression of collagen-degrading MMP-1, whose activity further hydrolyzes the ECM, thereby allowing GZMB to accomplish ECM remodeling indirectly and efficiently by mobilizing MMPs (69). Whether a similar mechanism operates in tumors remains an area for future investigation.

### 4.2 Regulation of epithelial-mesenchymal transition by GZMB

GZMB also plays a role in regulating the EMT in tumor cells. EMT involves epithelial cells acquiring mesenchymal characteristics through a series of molecular and morphological changes, which is essential for tumor invasion, metastasis, and drug resistance (70–72). D’Eliseo D et al. have identified a close association between GZMB and EMT in UC. GZMB is specifically expressed in cancer cells undergoing EMT at the tumor invasion front. These cells also exhibit positivity for Snail and N-cadherin, along with a lack of E-cadherin. As a critical molecule for maintaining epithelial cell adhesion and tissue integrity, the downregulation of E-cadherin can lead to cancer cell detachment, potentially enhancing invasion and metastasis (57). However, the specific mechanism by which GZMB influences EMT remains unclear. Previous studies suggest that EMT is regulated through multiple signaling pathways, with the TGF-β signaling pathway being a primary regulator. TGF-β activates both Smad-dependent and -independent pathways, which may be influenced by GZMB to induce EMT transcription factors such as Snail, Slug, and Twist. This results in the downregulation of the intercellular adhesion molecule E-cadherin and the upregulation of stromal markers such as N-cadherin and vimentin (73, 74). D’Eliseo D confirmed this influence in colorectal cancer cells, showing that GZMB plays a role in TGF-β1-induced EMT. Increased GZMB expression leads to upregulation of TGF-β1-driven EMT, while its reduction causes downregulation of TGF-β1-driven EMT (59). Although the link between GZMB and EMT has been preliminarily validated in colorectal cancer models, further research is necessary to determine its applicability to other tumor types and to clarify its specific role in the EMT process in human tumors.

### 4.3 Regulation of angiogenesis by GZMB

Tumor angiogenesis denotes the formation of new blood vessels within tumor tissue. These newly formed vessels supply tumor cells with essential oxygen and nutrients, thus promoting their growth. Furthermore, they facilitate the entry of these cells into the bloodstream, thereby enabling metastasis. Among the numerous factors involved, vascular endothelial growth factor (VEGF) serves as a pivotal regulator of tumor angiogenesis. VEGF, by binding to its receptors (VEGFR), activates downstream signaling pathways that enhance neovascularization (75, 76). Studies have shown that GZMB plays a regulatory role in angiogenesis. Extracellular GZMB can induce the release of VEGF from the ECM by cleaving fibronectin (77). Obasanmi et al. demonstrated that GZMB promotes angiogenesis through the upregulation of VEGF-A (78). Additionally, Wroblewski et al. identified a significant association between mast cells and resistance to anti-angiogenic therapies in tumors, noting that mast cells secrete substantial quantities of GZMB. This secretion, in turn, triggers the release of proangiogenic factors from the ECM, including FGF-1 and GM-CSF. These factors may bypass the targeted VEGFA-VEGFR2 signaling axis, promoting endothelial proliferation and angiogenesis even under anti-angiogenic treatment, thus reducing the efficacy of such therapies (53). Furthermore, Belfort-Mattos et al. observed that in cervical intraepithelial neoplasia (CIN), higher expressions of GZMB and VEGF were correlated with more severe lesions (79). The correlation between GZMB expression and VEGF upregulation suggests that GZMB may act as a critical nexus linking the TME to angiogenesis. This mechanism could not only facilitate tumor progression but might also contribute to the failure of anti-angiogenic therapies. It is important to recognize, however, that angiogenesis is governed by a complex and precise regulation of multiple factors. GZMB is likely only one of several pro-angiogenic contributors within the TME, and its specific functions warrant further investigation to confirm its roles and effects.

### 4.4 Regulatory roles of GZMB on effector T cells

GZMB has traditionally been considered a critical tool used by CTLs and NK cells to eliminate tumor cells. However, recent studies have expanded its recognized functions, demonstrating that it also exerts regulatory effects on effector T cells. GZMB inhibits the proliferation of these cells and promotes their apoptosis through multiple mechanisms, thereby playing a crucial role in the negative regulation of immune responses.

On one hand, GZMB indirectly suppresses T cell function and proliferation. Wieckowski E et al. demonstrated that the ζ chain of the TCR is a direct substrate for the proteolytic activity of GZMB, meaning that GZMB can hydrolyze this critical component of the TCR complex (80). The ζ-chain plays an essential role in T cell activation and signal transduction, and impairments in this chain are frequently associated with compromised immune functionality (81, 82). Furthermore, Lindner S and Fabricius D et al. have shown that GZMB inhibits T cell proliferation by degrading the TCR ζ-chain (42, 48). Although these findings were initially observed in pDCs and Bregs, further research is necessary to determine whether GZMB expressed by Tregs, MDSCs, or within solid tumors also exhibits similar TCR ζ-chain hydrolyzing activity. Moreover, studies indicate that GZMB can directly induce apoptosis in effector T cells. Cao X et al. discovered that GZMB derived from Tregs can induce apoptosis in both NK and CD8+ T cells via a perforin-dependent mechanism, thus impairing the tumor clearance capacity (29). Additionally, it has been shown that GZMB from Tregs can also kill effector T cells through a perforin-independent pathway, maintaining its cytotoxic activity even when perforin is inhibited (28, 30). Furthermore, Hoek KL et al. observed that GZMB within CD4+ T cells modulates their own differentiation; however, the implications of this process within tumors remain unvalidated, presenting a novel direction for future GZMB research (83).

## 5 The pro-tumorigenic role of GZMB in specific cancers

Although high expression of GZMB has traditionally been associated with a favorable prognosis owing to its anti-tumor functions, recent studies suggest a paradoxical correlation with adverse clinical outcomes, as summarized in **Table 2**.

**Table 2:** Pro-tumor effects of GZMB in specific cancers.

Cancer type	Expression	Proposed mechanisms	Refs
Androgen-Resistant Prostate Cancer (ARCaP)	Higher in Invasive ARCaP-M vs. Less Invasive ARCaP-E Phenotypes	Promotes Invasion	(86)
Urothelial Carcinoma (UC) & Bladder Cancer	Expressed in UC Tissue and Cell Lines	ECM Remodeling, EMT Regulation	(57)
Pancreatic Cancer	Expressed in PT45 Cell Line	Promotes Invasion	(58)
Colorectal Cancer (CRC)	Expressed in 57.1% of CRC Cell Lines and 100% of Cancer Stem Cells (CSCs)	ECM Remodeling, EMT Regulation	(59)
Nasopharyngeal Carcinoma (NPC)	Expressed in GZMB+ Tumor-Infiltrating Lymphocytes (TILs)	T-cell Exhaustion	(87)
Cervical Cancer	Expression Increases with Severity of Pre-cancerous Lesions (CIN)	VEGF Expression	(79)
Lymphoma (HD & ALCL)	Expressed in GZMB+ Cytotoxic T Lymphocytes	T-cell Exhaustion	(89)
Glioma	Expressed in GZMB+ Cytotoxic T Lymphocytes	T-cell Exhaustion	(90, 91)

Androgen-resistant cancer of the prostate (ARCaP) is characterized by a phenotype that is unresponsive to androgen therapy. Previous research has designated ARCaP as highly invasive and metastatic, associated with a poorer prognosis. ARCaP exhibits two phenotypes: ARCaP-E (epithelial phenotype) and ARCaP-M (mesenchymal phenotype), which serve as valuable models for studying EMT. The ARCaP-E phenotype displays a slightly reduced invasive capacity, whereas the ARCaP-M phenotype demonstrates a highly potent invasive capability (84, 85). Bou-Dargham MJ et al. conducted liquid chromatography-tandem mass spectrometry analyses of secreted proteins from these two phenotypic prostate cancer cell lines and discovered high GZMB expression in ARCaP-M and only weak expression in ARCaP-E. Knockdown of GZMB in ARCaP-M did not alter the expression of E-cadherin, N-cadherin, or vimentin but significantly diminished the invasive capacity of the tumor cells. This finding indicates that GZMB plays a crucial role in the invasion and metastasis of ARCaP, although the precise mechanisms remain to be elucidated (86).

D’Eliseo D et al. demonstrated that GZMB was highly expressed in multiple tumor types. Initially, using immunohistochemistry, they confirmed its expression in various bladder cancer cell lines and UC tissues while perforin was not expressed. Based on pathological features, there were significant differences in GZMB expression between high-grade and low-grade pTa tumors. Furthermore, its expression correlated significantly with tumor EMT. GZMB+ cells expressed Snail and N-cadherin but not E-cadherin. This suggests that GZMB is associated with tumor invasiveness, as knocking down or inhibiting GZMB expression significantly reduced tumor invasion. The researchers further assessed its function using bladder cancer cell lines and found that tumor-derived GZMB possesses enzymatic activity capable of cleaving fibronectin and remodeling the ECM. Consequently, GZMB promotes UC invasiveness through ECM degradation and remodeling, potentially serving as a marker for UC progression (57).

Subsequently, D’Eliseo D et al. validated GZMB expression in pancreatic cancer cell lines, discovering that the human pancreatic cancer PT45 cell line expresses it. Downregulating GZMB similarly significantly inhibited PT45 cell invasion. Interestingly, the researchers discovered that the n-3 polyunsaturated fatty acid docosahexaenoic acid (22:6n-3; DHA), an active derivative of fish oil, dose-dependently downregulated GZMB in human pancreatic cancer PT45 cells and human bladder cancer RT112 cells. Thus, DHA reduces the invasiveness of bladder and pancreatic cancer cells (58).

D’Eliseo et al. conducted further analyses on GZMB expression in seven CRC cell lines and four patient-derived cancer stem cells (CSCs). They discovered that GZMB was expressed in 57.1% of the CRC cell lines and in 100% of the CRC-derived CSCs, with extracellular secretion also occurring. The knockdown of GZMB in invasive CRC cells led to a reduction in their invasive capacity. Additionally, the analysis of three EMT biomarkers, Snail1, E-cadherin, and N-cadherin, demonstrated that GZMB downregulation suppressed TGF-β1-driven EMT. Consequently, they proposed that tumor-associated GZMB promotes cancer invasion and EMT. Similarly, they observed that DHA inhibited its expression and tumor invasiveness in CRC cells *in vitro*, suggesting its potential as an anti-tumor agent (59).

In pathological samples from nasopharyngeal carcinoma patients, numerous GZMB+ tumor-infiltrating lymphocytes (TILs) were identified. Researchers found that higher percentages of GZMB+ TILs were associated with shorter PFS and OS, with the prognostic value of GZMB-positive TIL counts surpassing that of T and N staging. The vast majority of these cells were CD8+ and CD56-, indicating that most GZMB+ TILs represent activated CTLs. These findings suggest that tumor cells may evade CTL-induced cell death, especially in nasopharyngeal carcinoma patients with poor prognoses (87).

As early as 2008, a six-month prospective study on cervical cancer revealed that GZMB expression was significantly associated with poor treatment response and unfavorable prognosis (88). Belfort-Mattos et al. investigated the relationship between the severity of CIN and GZMB expression, revealing higher expression with increasing lesion grade. Concurrently, VEGF expression levels also rose with lesion progression. This coordinated upregulation suggests a potential role for GZMB in modulating the local immune microenvironment and promoting angiogenesis during neoplastic progression (79).

In hematological malignancies, ten Berge RL et al. (2001) noted that a high proportion of activated CTLs might portend a poor prognosis. Under normal conditions, CTLs express both TCR and CD8, yet they lack cytotoxic capacity. It is only upon receiving crucial signals that CTLs become activated, notably expressing high levels of GZMB, perforin, and CD25, and subsequently acquire proliferative and cytotoxic capabilities. In their study, researchers identified a high proportion (≥15%) of activated CTLs in biopsy specimens from patients with Hodgkin’s disease and ALK-negative anaplastic large cell lymphoma (ALCL), which correlated with poorer OS and PFS (89). This outcome raises a paradoxical question: why does a high proportion of activated CTLs correlate with poorer prognosis? This observation suggests that the mere number of CTLs does not necessarily reflect their functional efficacy. Despite the accumulation of large numbers of activated CTLs in the TME, these cells often exhibit functional impairment or exhaustion.

In the context of gliomas, Cui X et al. identified that the Runt-related transcription factor 1 (RUNX1) mediates ECM remodeling and promotes the formation of an immunosuppressive TME, thus facilitating tumor progression. ELISA tests showed that tumors with elevated RUNX1 levels also displayed higher GZMB concentrations, hinting at a potential role for GZMB in this pathway. Nevertheless, the specific mechanisms by which GZMB influences these processes require further elucidation (90). In a separate multi-omics study concerning glioma, Wischnewski et al. reported the presence of abundant GZMB-producing effector T cells, which simultaneously exhibited high levels of various immune checkpoint molecules, such as PD-1 and TIM-3. This profile of co-expression suggests a state of functional dysfunction or exhaustion, which ultimately impairs their cytotoxic functions (91).

Accumulating evidence suggests that GZMB plays a highly complex and dualistic role in tumor progression. In various cancers, increased GZMB expression correlates significantly with tumor invasion, metastasis, and poor prognosis. This association stems primarily from two factors. First, tumor cells themselves may express GZMB, which directly degrades ECM components such as fibronectin, promoting EMT and angiogenesis, thus enhancing the tumor’s invasive and metastatic capabilities. Second, a high proportion of GZMB produced by CTLs in the TME does not necessarily indicate an effective immune response but may reflect T cell dysfunction or exhaustion. Although these CTLs are capable of producing GZMB, their overall cytotoxic activity is diminished by immunosuppressive molecules such as PD-1 and TIM-3, leading to immune escape. It is critical to recognize that current research does not definitively establish GZMB as a direct cause of poor prognosis; rather, other molecular or cellular processes, still undetected, might play significant roles, necessitating further mechanistic studies. Moreover, the TME constitutes a highly intricate system where the pro-tumorigenic role of GZMB does not occur in isolation but in conjunction with various cells, cytokines, and chemokines, whose interrelations within this network remain largely unexplored.

## 6 The determinants of GZMB’s dual function in cancer

GZMB, as the primary effector molecule in CTLs and NK cells, has been traditionally recognized for its anti-tumor properties through the induction of target cell apoptosis. Recent extensive research, however, has uncovered its non-classical roles in tumor development, illustrating a complex dual role that both enhances anti-tumor immunity and potentially facilitates tumor immune evasion and progression.

GZMB’s role in tumor immunity is exceptionally complex and contradictory. Its ultimate impact, whether anti-tumor or pro-tumor, depends primarily on its source, site of action, and target cell, as summarized in **Table 3**. When GZMB is derived from CTLs and NK cells, it enters target cells via perforin-dependent pathways or perforin-independent pathways (such as endocytosis) to exert its effects intracellularly. Initially, GZMB activates the caspase cascade to induce apoptosis in target cells. Additionally, it can cleave other intracellular substrates, such as Bid and ICAD, leading to mitochondrial dysfunction and DNA damage, thereby promoting caspase-independent apoptosis (3). Conversely, when GZMB is sourced from Tregs, MDSCs, pDCs, or solid tumor cells, it predominantly functions extracellularly. In these scenarios, it promotes tumor progression by cleaving ECM proteins, which disrupts tissue integrity and facilitates metastasis; impairing T cell function by cleaving surface molecules like the TCR, thereby inhibiting proliferation and enabling immune escape; and modulating cytokines such as VEGF, FGF-1, GM-CSF to stimulate tumor neovascularization (5, 21).

**Table 3:** The determinants of GZMB’s dual role in cancer.

Determinant	Anti-tumor effects	Pro-tumor effects
Cellular Source	CTLs, NK cells	Tregs, Bregs, pDCs, MDSCs, mast cell, tumor cells
Localization & Context	Delivered into the cytoplasm of target cells via perforin	Released into the ECM, acts intracellularly within immune cells
Substrate	apoptotic substrates (caspases, Bid)	immunoregulatory molecules (TCR zeta-chain) or ECM components
Function	Tumor cell apoptosis	ECM remodeling, EMT Regulation, Angiogenesis, T cell proliferation and apoptosis

Moreover, the concentration of GZMB significantly influences its efficacy. Ida H et al. observed that GZMB, upon CD2 stimulation, leaked intracellularly from NK cells, escaping from cytotoxic granules into the cytoplasm where it cleaves Bid, triggering self-apoptosis. NK and CTL cells inherently possess PI-9 to prevent self-damage. Therefore, a leakage of GZMB exceeding PI-9 levels may be a decisive factor in cell death (92–94). Beyond intracellular leakage, extracellular leakage may occur as well. When CTL or NK cells undergo sustained activation, excessive production of GZMB may overwhelm the delivery capacity of the ‘immune synapse’, leading to its leakage into the ECM, where it may exert alternative effects. Consequently, the function of GZMB is dynamic and complex. An essential direction for future research is to elucidate the precise thresholds and mechanisms that underlie its transition from anti-tumor to pro-tumor effects within the TME. This exploration will provide a critical theoretical foundation for developing novel strategies for the targeted delivery of GZMB.

## 7 The clinical translation of GZMB

GZMB, a critical effector molecule in tumor immune responses, has attracted considerable interest for its potential in clinical translation. As the principal mediator of target cell apoptosis, its utilization in tumor therapy has emerged as a significant area of research. Recent studies have investigated the targeted delivery of GZMB using nanomaterials for treating gastric cancer and lymphoma (19, 95). Although these studies have yielded promising results in animal models, several challenges impede their translation to clinical settings. Initially, research has predominantly concentrated on the cytotoxic effects of GZMB. Yet, the role of GZMB extends beyond merely inducing intracellular death; it also affects other processes such as ECM degradation and the regulation of angiogenesis within the TME. Even if targeted delivery of GZMB succeeds in eliminating certain tumor cells, the suppressive nature of the TME might inhibit effective immune responses, potentially leading to drug resistance. Moreover, tumor cells frequently express PI-9, a natural antagonist of GZMB, which binds to its active site and completely neutralizes it, thereby nullifying its tumor-killing effect (96, 97). High levels of PI-9 expression in tumors may thus represent a key mechanism by which tumors evade immune surveillance, contributing to resistance against CTL-mediated destruction and to poorer prognoses. Huang H et al. have shown that delivering chemotherapy drugs in conjunction with PI-9 inhibitors directly to tumor cells enhances the efficacy of treatments for pancreatic cancer (98). This discovery points towards a novel research direction where both GZMB and PI-9 inhibitors could be precisely targeted to tumor cells. Nevertheless, this strategy poses significant challenges, as off-target effects may damage healthy tissues. A more feasible approach may involve the development of tumor-targeting PI-9 inhibitors that could be used in combination with existing immunotherapies to effectively activate or recruit effector T cells. This dual approach has the potential to simultaneously overcome tumor resistance and enhance tumor-killing efficacy.

It has been noted that activated CTLs may express inhibitory receptors at high levels, entering a state of “functional exhaustion.” Despite elevated GZMB expression, these CTLs may remain ineffective in exerting anti-tumor effects and could be associated with a poorer prognosis (90, 91). Therefore, the development of therapies that combine GZMB with immune checkpoint inhibitors or nanodelivery systems could significantly improve the effectiveness of tumor immunotherapy.

Chimeric antigen receptor T-cell immunotherapy (CAR-T therapy) represents a cutting-edge approach in tumor immunotherapy. Through genetic engineering, this method adapts a patient’s own T cells to accurately recognize and effectively eradicate cancer cells. It is currently authorized for the treatment of hematological malignancies (99–101). Yet, in the context of solid tumors, the TME presents significant obstacles to the efficacy of CAR-T therapy (102, 103). The ζ chain plays a pivotal role as a crucial structural element of the CAR in CAR-T cell therapy, tasked with relaying essential signals necessary for T-cell activation. Prior research has established that GZMB can hydrolyze the ζ chain (42, 48), which interrupts the signaling required for T cell activation. This process likely contributes to the functional exhaustion of CAR-T cells and subsequent treatment failure, marking a significant factor that affects the efficacy of CAR-T therapy. However, there remains a paucity of direct evidence linking GZMB-mediated hydrolysis of the ζ chain to compromised CAR-T cell function, indicating an important area for future investigation.

In summary, GZMB serves as a crucial effector molecule in tumor immunity and holds considerable therapeutic promise. Nevertheless, its translation into clinical practice faces numerous hurdles. Although current strategies employing nanotechnology for GZMB delivery have shown some success, their effectiveness remains curtailed by the immunosuppressive nature of the TME. Furthermore, tumor cells frequently overexpress their endogenous inhibitor, PI-9, which facilitates immune evasion and is associated with an adverse prognosis. This observation suggests that integrating immunotherapies with PI-9 inhibitors might augment anti-tumor responses. Moreover, despite high levels of GZMB expression by CTLs, T-cell exhaustion can still limit therapeutic success. Therefore, there is a pressing need to develop GZMB-based combination therapies, including immune checkpoint inhibitors, to improve treatment outcomes. In CAR-T therapy, the GZMB-mediated hydrolysis of the ζ chain could result in interruption of signal transduction and T-cell dysfunction, potentially representing a critical mechanism that limits the efficacy of CAR-T in solid tumors, although further direct evidence is essential for confirmation. Given its dual roles within the TME, more research is needed to selectively enhance its anti-tumor activity while mitigating its immunosuppressive effects.

## 8 Conclusion

In summary, GZMB displays a paradoxical role in tumor initiation and progression. On one hand, it serves as a cytotoxic effector molecule that promotes tumor cell apoptosis and facilitates immune clearance. On the other hand, it contributes to tumor progression by modulating the TME, remodeling the ECM, and influencing the function of immunosuppressive cells. Its established cytotoxic function supports tumor immunotherapy, whereas its non-classical actions foster immune evasion and further tumor development. Consequently, strategically harnessing its beneficial effects while mitigating its detrimental impacts remains a critical research objective. Theoretically, one strategy could involve the development of nanomedicines containing GZMB, which should be precisely targeted to tumor cells and engineered to activate their cytotoxic effects solely upon cellular entry. An alternative strategy might focus on developing inhibitors of GZMB that are highly specific, aiming to inhibit extracellular GZMB to block its pro-tumoral activities without impairing its intracellular cytotoxic functions. However, it is imperative to acknowledge that any therapeutic approach targeting GZMB activity entails significant risks, as it could simultaneously disrupt the essential antitumor functions of CTLs and NK cells, thus compromising immune surveillance and reducing therapeutic viability. Nonetheless, this challenge does not eliminate the possibility of targeting GZMB’s non-canonical functions. Future research should focus on differentiating between the canonical and non-canonical roles of GZMB, elucidating the mechanisms that underlie GZMB’s functional shifts within various TMEs, and establishing a robust theoretical basis for devising precise and efficacious therapeutic interventions.
